# Unusual Presentation of a Sphenoidal Sinus Neuroendocrine Tumor: A Case Report and Review of Literature

**DOI:** 10.7759/cureus.13689

**Published:** 2021-03-04

**Authors:** Jasmeet Kaur, Swathi Mogulla, Ambreen Malik, Sandeep Garg

**Affiliations:** 1 Internal Medicine, Saint Joseph Mercy Oakland Hospital, Pontiac, USA; 2 Hematology and Medical Oncology, Saint Joseph Mercy Oakland Hospital, Pontiac, USA

**Keywords:** paranasal sinus neoplasm, sphenoid sinus, antineoplastic combined treatment protocol, electron and proton beam therapy, etoposide, neuroendocrine carcinoma (nec)

## Abstract

Neuroendocrine tumors (NETs) have a heterogeneous pathology and indolent behavior, with the most common location being the gastrointestinal tract and then the lungs. The head and neck are rare sites of NET presentation with varied clinical signs and symptoms, which occasionally delay the diagnosis, thereby leading to an advanced stage at presentation. We present a rare case of paranasal sinus small cell neuroendocrine tumor and perform a review of the literature. A 71-year-old man presented with continuous bleeding from the left nostril and nasal congestion without any prior medical history. Clinical examination revealed nasal congestion, rhinorrhea, postnasal drip, and active bleeding. The laboratory data were within normal limits. Imaging studies showed a left sphenoid sinus mass extending to the left ethmoid sinus with a break in the cribriform plate encephalocele. An enlarged lymph node measuring 2.2 cm was noted in the left neck and supraclavicular region. The evaluation through stereotactic endoscopic sinus surgery and biopsy revealed left nasopharyngeal, sphenoid sinus, and ethmoid sinus masses. Pathologic biopsy findings were consistent with high-grade, malignant, small, round blue cell tumors. Immunohistochemical analysis confirmed high-grade small cell neuroendocrine carcinoma. The patient was diagnosed with stage IV (TXN2bM0, T: tumor size, N: lymph node, M: metastasis) high-grade neuroendocrine tumor of the left paranasal sinus. He was treated with concurrent chemoradiation therapy with two cycles of etoposide and cisplatin and radiation therapy with proton beam radiation therapy followed by two cycles of adjuvant etoposide cisplatin chemotherapy. The patient showed a good response to the treatment, as confirmed using imaging. He is currently being regularly followed up with serial imaging.

## Introduction

Neuroendocrine neoplasm (NEN) is a group of malignancies known for their heterogeneity and indolent behavior [1]. These rare tumors arise from neuroendocrine/enterochromaffin cells and their progression and prognosis are highly dependent on the anatomic location [1]. According to the United States Cancer Statistical Analysis, from 2001 to 2015, the incidence of NEN was 2.89 per 100,000 people annually [2]. The common sites of NEN are the tubular gastrointestinal tract and bronchopulmonary system; however, it can present in other rarer sites, including the head and neck, urinary bladder, and ovaries [3]. NEN is categorized based on cellular differentiation. A well-differentiated NEN is termed a neuroendocrine tumor (NET), whereas a poorly differentiated NEN is termed a neuroendocrine carcinoma (NEC) with small or large cell types [4]. Small cell neuroendocrine carcinoma (SmCC) is a high-grade and aggressive tumor with a high potential for recurrence and metastasis [5]. SmCC most commonly presents as primary pulmonary neoplasms near the bronchial tree in up to 25% of patients and are uncommon in the head and neck region [6]. NEN of the head and neck are rare tumors characterized as the SmCC type [7]. The latter is commonly found in the larynx, followed by the sinonasal region and salivary glands [8]. Though the incidence of sinonasal SmCC is uncertain, approximately 60-120 cases of the head and neck are presented for every 1000 extrapulmonary small cell cancers diagnosed annually in the United States [9]. SmCCs present with vague symptoms of nasal obstruction, nasal drainage, and epistaxis; hence, they present with advanced disease [8]. The most common sites of involvement, in order of occurrence, is ethmoid sinus, nasal cavity, and maxillary sinuses [8]. Most patients with sinonasal NETs present with advanced-stage disease. The surgery is curative for patients with localized disease but not in advanced-stage disease. The chemotherapy regimen for the treatment of advanced-stage SmCC is platinum or etoposide-based regimens similar to that used for high-grade NECs of the lung, as both have similar histopathologies.

We present a rare case of paranasal sinus SmCC, along with a literature review for the diagnosis and management of paranasal sinus NEC.

## Case presentation

**Figure 1:**
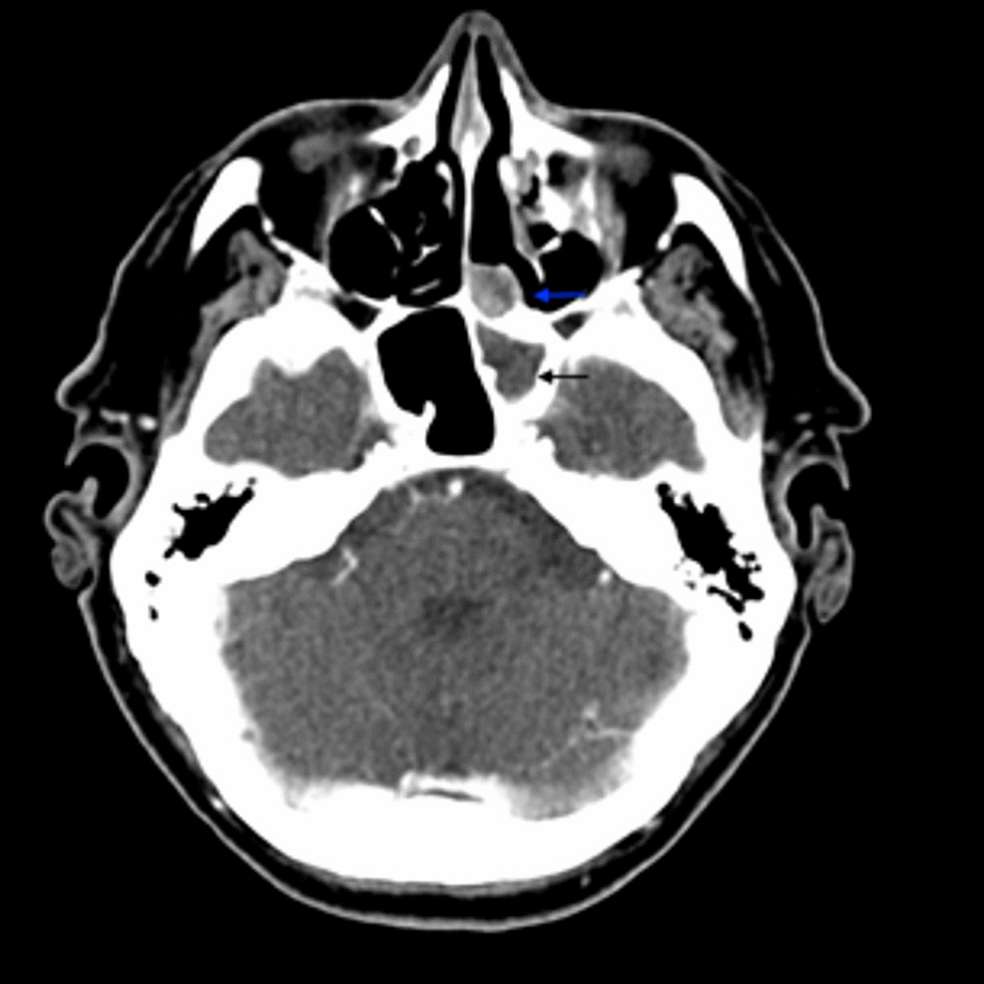
Axial view of contrast-enhanced CT showing a soft tissue density structure causing complete opacification of the left sphenoid sinus and extension into the adjacent ethmoid sinus(as demonstrated by black and blue arrows, respectively CT: computed tomography

**Figure 2:**
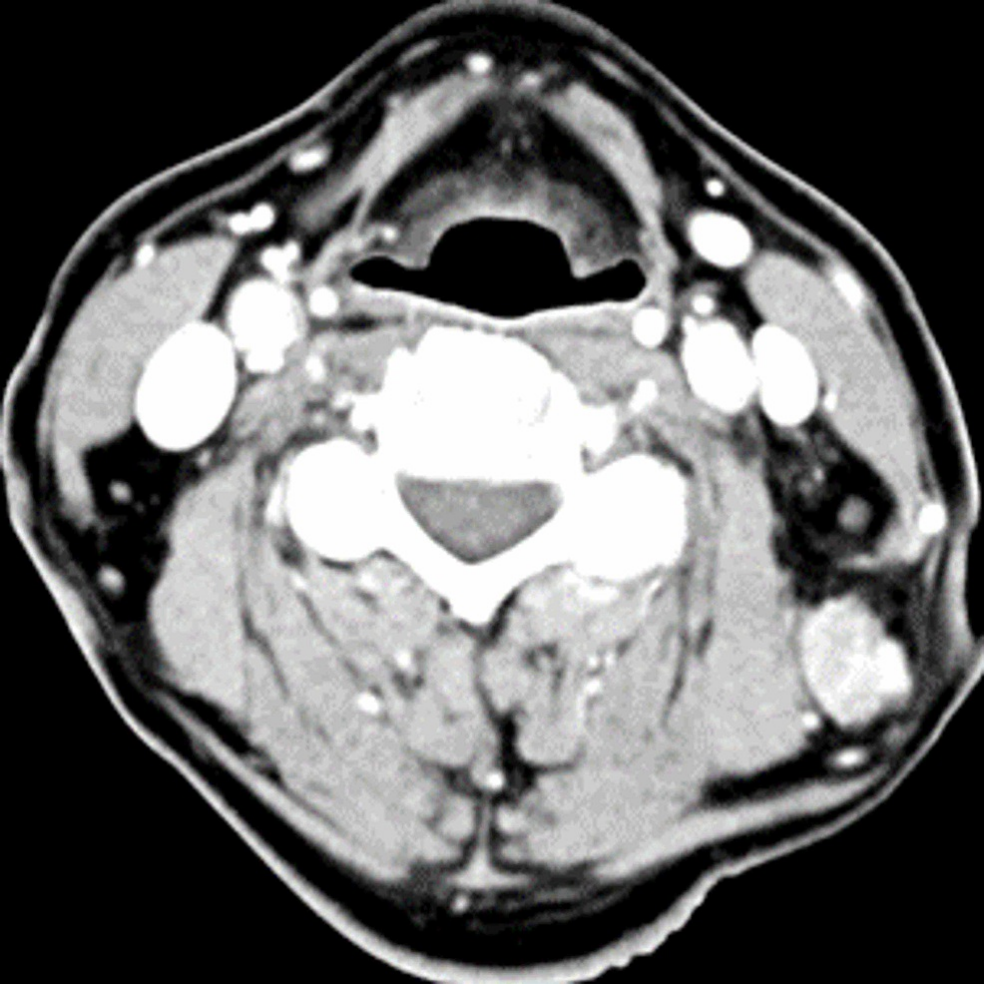
CT head and neck axial view thin sections demonstrating nodal metastasis with pathologically enlarged level 3 and 5 lymph nodes, that is, the posterior upper jugular and upper posterior triangle lymph nodes CT: computed tomography

**Figure 3:**
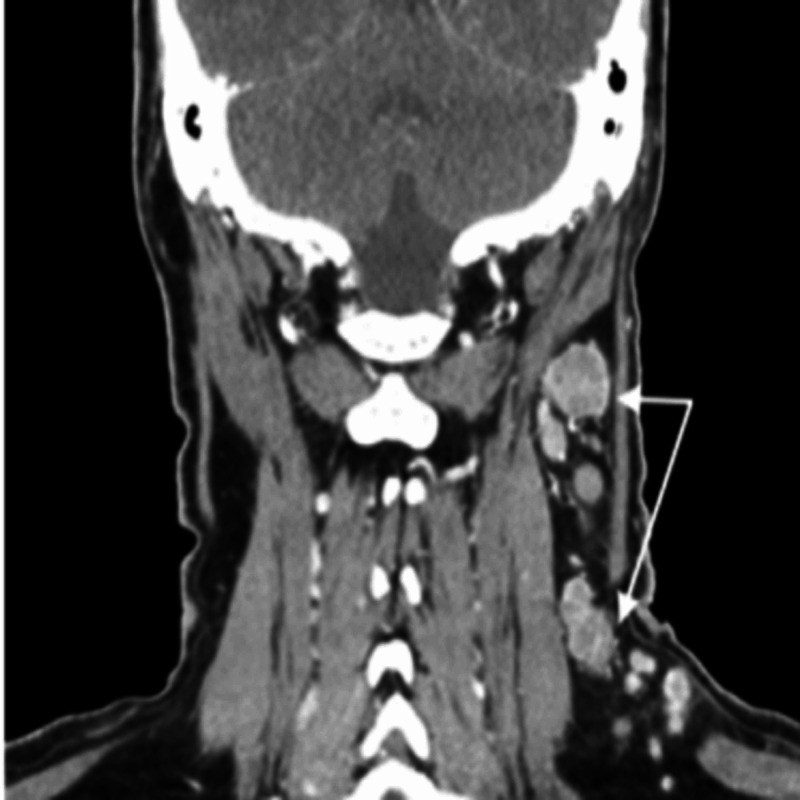
Coronal section of CECT demonstrating enlarged left upper posterior and mid jugular lymph nodes. Some of the nodes have central hypo enhancement with peripheral ring enhancement suggesting a necrotic process (marked with white arrows) CECT: contrast-enhanced computed tomography

**Figure 4:**
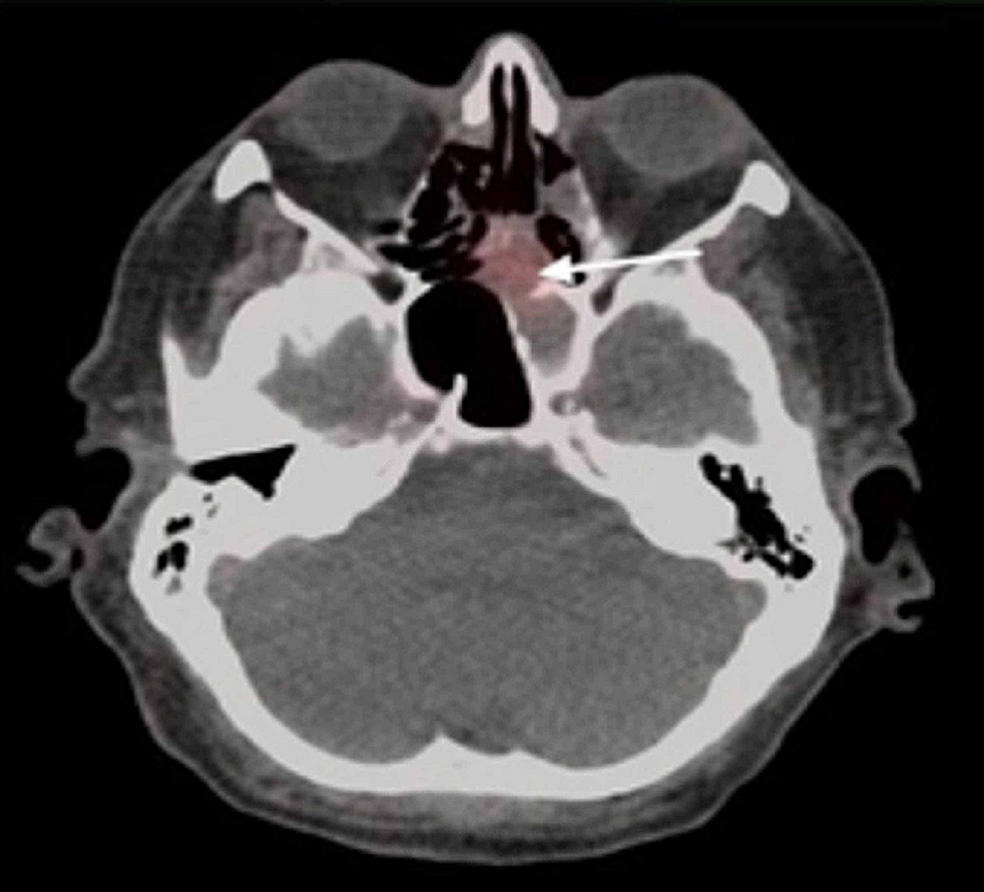
FDG PET CT shows increased uptake in the soft tissue structure that extends from the sphenoid sinus into the ethmoid sinus which can be secondary to neoplastic or inflammatory process. But since this patient has pathologically enlarged lymph nodes, a neoplastic process cannot be excluded. Tissue sampling was indicated for further evaluation. FDG: fludeoxyglucose, PET: positron emission tomography, CT: computed tomography

**Figure 5:**
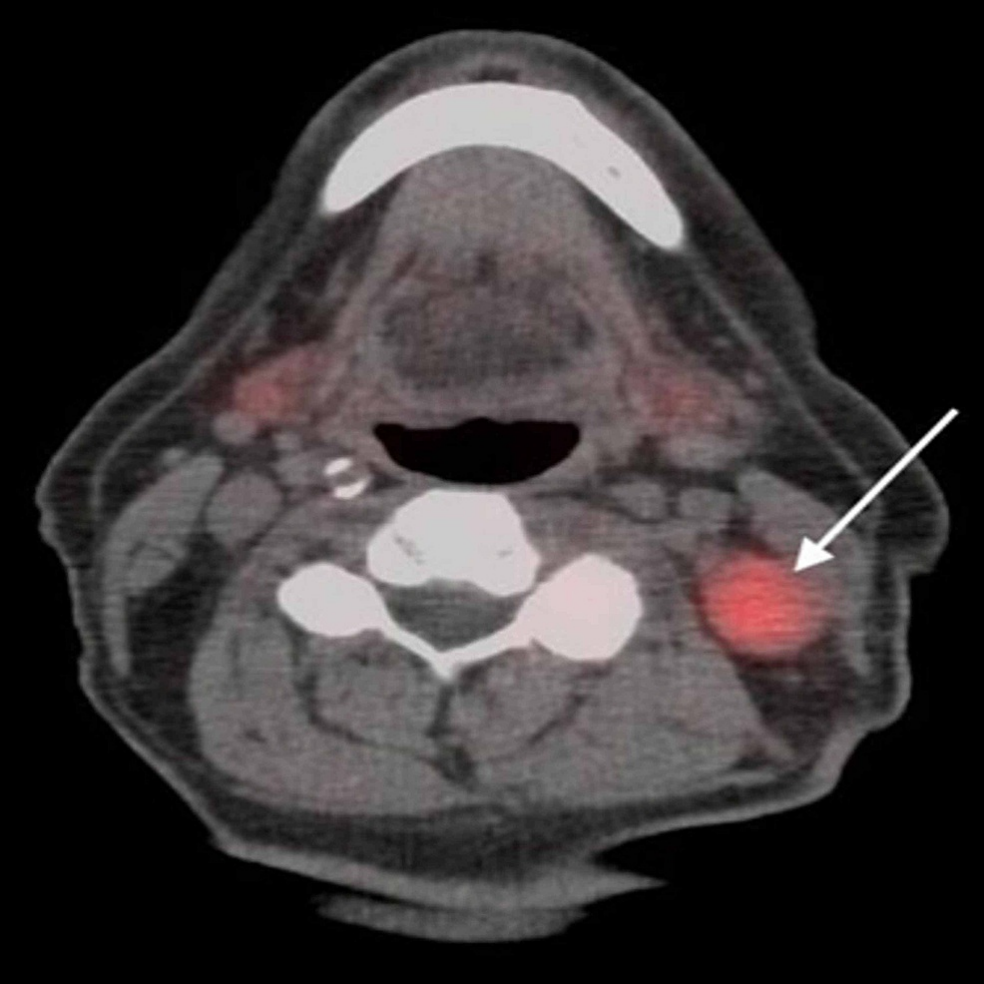
FDG-PET-CT axial view demonstrating increased uptake in the enlarged left upper jugular (posterior) lymph node FDG: fludeoxyglucose, PET: positron emission tomography, CT: computed tomography

A 71-year-old man with an unremarkable medical history presented to the clinic with complaints of continuous bleeding from the left nostril for nine to 10 days associated with irregular nasal congestion. The patient denied a history of trauma, nose-picking, difficulty in nasal breathing, facial swelling, sinus pressure, sore throat, eye redness or watering, cough, difficulty breathing, and chest pain. He denied ear pain, ear fullness, change in hearing, dizziness, syncope, tinnitus, difficulty in swallowing, change in appetite, weight change, nausea, vomiting, blood in urine, dark stool, hematemesis, bleeding gums, bruises, or skin rash. He had a 50 pack-year smoking history with no family history of cancer. Clinical examination revealed congested nasal turbinate with active bleeding, without any identifiable mass or ulcer. The laboratory data on presentation included were white blood cell count 9000 (4000-10,000)/µL, hemoglobin 15 (13-17) g/dL, platelets 219 (140-400) K/µL, blood urea nitrogen 15 (7-20) mg/dL, and creatinine 0.89 (0.5-1.3) mg/d; he tested negative for HIV, hepatitis C, and hepatitis B. Computed tomography (CT) of the neck, and paranasal sinuses showed a left sphenoid sinus mass extending to the left ethmoid sinus with a break in the cribriform plate encephalocele (Figure 1). There were enlarged lymph nodes noted at the left posterior upper, mid, and lower jugular level (Level 3 and 4a) and posterior supraclavicular region (Level 5a), with the largest lymph node measuring 2.2 cm (as seen in Figures 2-3). This was further evaluated with fludeoxyglucose (FDG)-positron emission tomography (PET) CT that showed increased uptake in the sigmoid and ethmoid sinus and left neck lymph nodes with no increased uptake in the lungs, liver, and bone (Figures 4-5). The evaluation using stereotactic endoscopic sinus biopsy by the otolaryngologist revealed left nasopharyngeal, sphenoid sinus, and ethmoid sinus masses.

**Figure 6:**
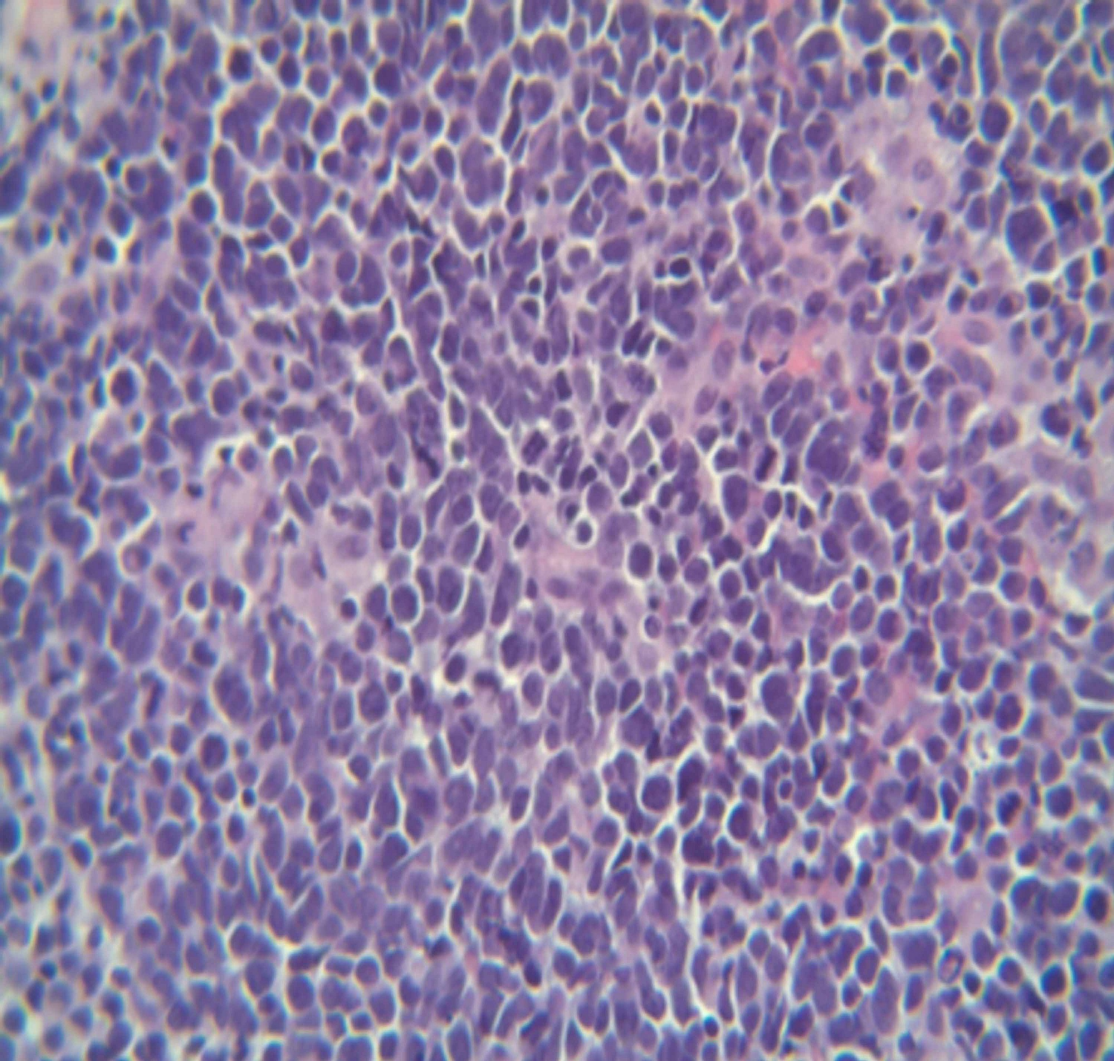
Small cell neuroendocrine carcinoma under 40x magnification. Positive for cytokeratin, synaptophysin, and chromogranin. Immunomorphological features are diagnostic of high-grade small cell neuroendocrine carcinoma

**Figure 7:**
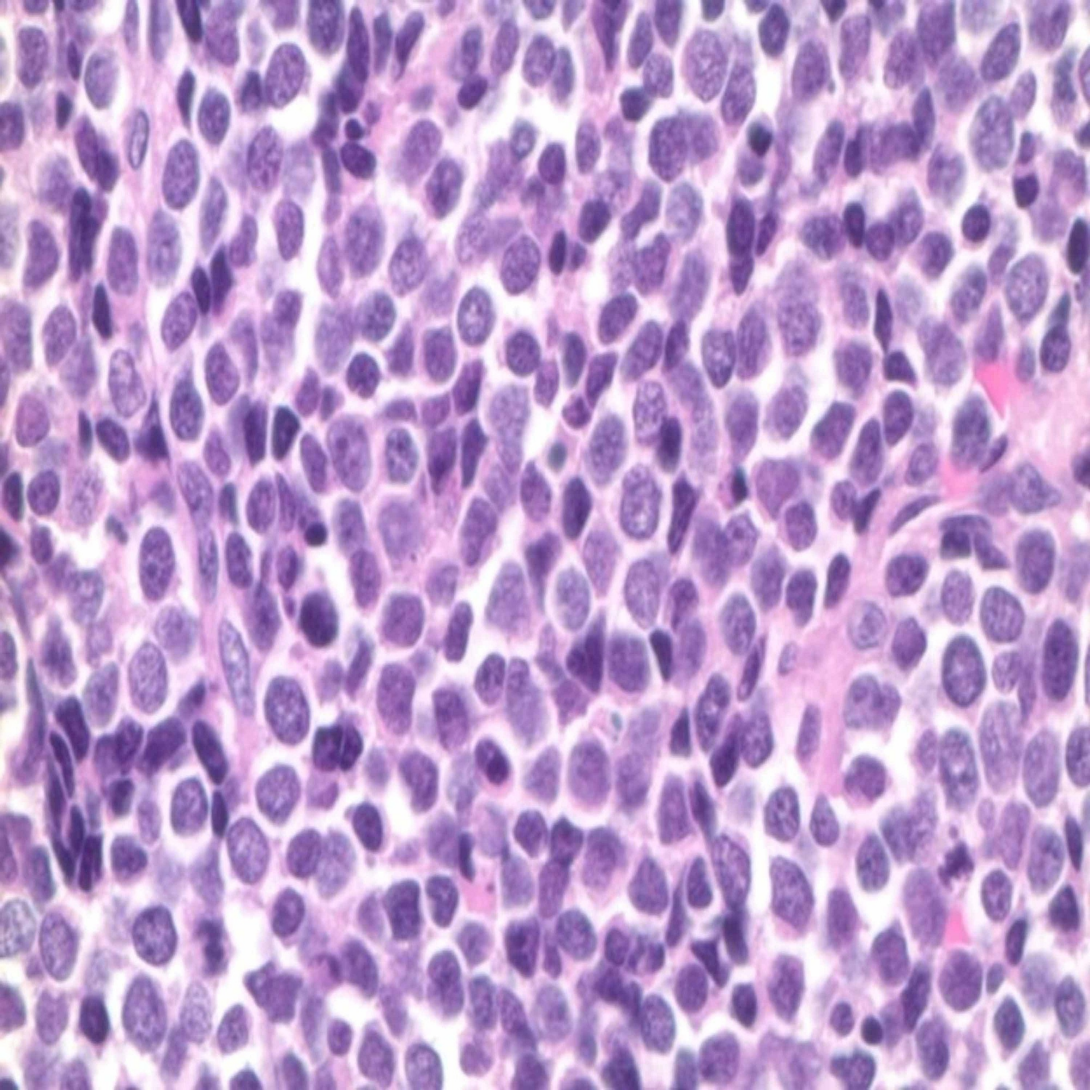
Small cell neuroendocrine Sonoma under 40x magnification. Immunostain was positive for keratin stain confirming the epithelial nature of the cell. Cells were also diffusely positive for synaptophysin

Biopsy findings were consistent with a high-grade, malignant, small, round blue-cell tumor (Figures 6-7). Immunohistochemically, the tumor was positive for the expression of cytokeratin, synaptophysin, and chromogranin; however, it was negative for the expression of thyroid transcription factor-1 (TTF1) desmin, cluster of differentiation (CD) 20, and CD99, consistent with high-grade SmCC. The Eastern Cooperative Oncology Group performance status was 0, and his condition was diagnosed as stage IV (TXN2bM0) high-grade neuroendocrine tumor of the left paranasal sinus. The patient’s case was presented to the hospital's tumor board as guidelines for treating high-grade stage IV neuroendocrine tumors of the paranasal sinus are lacking. The patient was treated with concurrent chemoradiation therapy with two cycles of etoposide and cisplatin and concurrent radiation therapy with proton beam radiation therapy followed by two adjuvant etoposide cisplatin cycles of chemotherapy. The patient showed a good response to the treatment, as confirmed using PET imaging. He developed deep vein thrombosis of the common femoral vein and thrombosis of the superficial vein. Treatment using a long-term oral anticoagulant, Eliquis, was initiated. He is being regularly followed up with serial imaging.

## Discussion

A NET in the paranasal sinus is rare [10]. Sinonasal tumors comprise 5% of head and neck cancer. The most common pathology being squamous cell carcinoma (56%) and adenocarcinoma (12%) [10]. There were 1024 patients with small cell cancer of the head and neck identified in a national database study [11]. Eighty-three percent of patients have been diagnosed with stage III-IV cancer, with approximately 30% tumors located in the nasal cavity and paranasal sinus [11]. For sinonasal NET, the tumor location was usually the ethmoid sinus, nasal cavity, and maxillary sinus [10]. NET arises from enterochromaffin cells, which stain with potassium chromate due to serotonin [7]. NET cells produce abundant neurosecretory amines such as synaptophysin and chromogranin. Some tumors may also secrete specific peptide hormones or bioamines (such as insulin, glucagon, somatostatin, vasoactive intestinal peptide, serotonin, and gastrin) that result in clinical syndromes [7]. SNEC tumors have rare, heterogeneous histopathology, with an ambiguous clinical course and prognosis [12]. The World Health Organization (WHO) classification for NET is based on cell differentiation, grade, and mitotic index (Table 1) [13]. SNEC is characterized by frequent local recurrence (45%) and distant metastasis (35%) despite administering multimodal therapy [8]. The most common sites of distant metastasis in NETs are the cervical lymph nodes, lung, liver, bone marrow, and vertebrae [8]. The determination of the location and local extension of SNEC is interpreted with imaging studies such as CT and MRI [8].

**Table 1:** WHO 2019 updated classification of NET Source: [13] NET: neuroendocrine tumor, G: grade, NEC: neuroendocrine neoplasm, MiNEN: mitotic neuroendocrine neoplasm, WHO: World Health Organization

Terminology	Differentiation	Grade	Mitotic rate (mitoses/2mm2)	Ki-67 index (percent)
NET, G1	Well-differentiated	Low	<2	<3
NET, G2	Well-differentiated	Intermediate	2-20	3-20
NET, G3	Well-differentiated	High	>20	>20
NEC, small cell type (SCNEC)	Poorly differentiated	High	>20	>20
NEC, large cell type (LCNEC)	Poorly differentiated	High	>20	>20
MiNEN	Well or poorly differentiated	Variable	Variable	Variable

A prospective single-arm trial by Fitzek et al. enrolled 19 patients with olfactory neuroblastoma and NEC [14]. The patients received two cycles of neoadjuvant chemotherapy with etoposide and cisplatin, followed by radiation treatment using high-beam proton therapy. Patients who responded to the treatment received two cycles of adjuvant chemotherapy. The study showed a five-year survival rate of 74%, and the local disease control rate at five years was 88% [14]. A meta-analysis of 701 published cases of sinonasal NEC by Van der Laan et al. highlighted the importance of the differentiation grade as a prognostic indicator and determining treatment strategies [15]. They developed a classification system for sinonasal NEC (Table 2) to better understand and streamline the treatment strategies for sinonasal NEC. The meta-analysis showed that of the 701 cases, 127 were well-differentiated sinonasal neuroendocrine carcinomas (SNEC), 459 were sinonasal undifferentiated carcinomas (SNUCs), and 115 were sinonasal small cell carcinomas (SmCCs). The five-year disease-free survival (DFS) was significantly different based on tumor histopathology (70.2% for SNEC, 35.9% for SNUC, and 46.1% for SmCC; p < .001). Surgical treatment improved the five-year DFS at 52.2% vs 30.1% in people who did not have surgery, p-value <0.001. The combination of radiotherapy and surgery improved outcomes compared with those obtained using radiotherapy alone (5-year DFS, 54.7% vs. 15.7%; p = .027). Monotherapy with chemotherapy alone in these patients had worse outcomes [15].

**Table 2:** Classification of sinonasal neuroendocrine tumor Source: [15] SNEC: sinonasal neuroendocrine carcinoma, SmCC: sinonasal small cell carcinoma, SNUC: sinonasal undifferentiated

Legacy terminology	Abbreviation	Differentiation grade	Cell size	Proposed terminology
Carcinoid	SNEC	Well	-	Grade I
Atypical carcinoid	SNEC	Moderate	-	Grade II
Small cell neuroendocrine carcinoma	SmCC	Poor	Small	Grade IIIA
Sinonasal undifferentiated carcinoma	SNUC	Poor	Moderate to large	Grade IIIB

A retrospective review of eight patients with high-grade SmCC showed improved outcomes in patients receiving multimodal treatment with surgery, radiotherapy, and chemotherapy when compared with monotherapy alone [16]. A retrospective case series of 37 patients with sinonasal malignancies with neuroendocrine differentiation showed 14 cases with SNUC, 14 with esthesioneuroblastoma (ENB), and nine with SNEC [17]. All patients with high-grade SNUC and SNEC received neoadjuvant chemotherapy followed by surgery and locoregional radiation therapy. While half of the patients with ENB had high grades, the tumor was treated with upfront surgery followed by locoregional radiation therapy. Follow-up data were available for only 16 patients (five SNUC, four SNEC, and seven ENB) with a mean follow-up of 11.5 (range, 2-56) months. Three patients with SNUC and four with SNEC developed locoregional recurrence at five and eight months, respectively. One patient with SNUC and one with SNEC developed distant metastasis at eight and four months, respectively [17].

A national population-based study by surveillance epidemiology showed that the prognosis of small cell carcinoma of the head and neck is poorer than that of squamous cell carcinoma [18]. According to the national cancer database survey, nasal cavity and paranasal sinus tumors have the best prognoses compared with other head and neck small cell cancer locations. In locally advanced disease, no difference was found between combined treatment with surgery, radiotherapy, and chemotherapy versus radiotherapy and chemotherapy [11].

A small case series of 12 patients with non-metastatic small cell carcinoma of the head and neck concluded that radiotherapy with and without chemotherapy provided a reasonable treatment alternative to surgery [19].

Our patient presented with minor nasal bleeds and was found to have a sphenoid mass extending to the ethmoid sinus and cribriform plate. Biopsy showed high-grade malignant small cell neuroendocrine tumors associated with supraclavicular lymph node involvement. The sphenoid sinus is an infrequent presenting location, and the incidence of the tumor in the location has not been reported in the literature. Our patient was treated with platinum-based chemotherapy as per the small cell carcinoma guidelines in conjunction with proton beam radiation. The preferred treatment strategy in most SNEC cases with poorly differentiated subtypes is surgery followed by radiotherapy [8]. Owing to the rarity of cases, there are no prospective trials to determine the appropriate treatment strategies in these patients [15]. In sinonasal small cell carcinoma, treatment with local radiation therapy and chemotherapy is a better alternative to surgery. The tumor’s unapproachable location makes it challenging to achieve negative resection margins [11,18-19].

Surgery is considered the cornerstone of treatment for NET. However, radiotherapy and chemotherapy are alternative approaches in treating tumors such as sphenoid sinus small cell carcinoma.

## Conclusions

Effective differentiation of SmCC tumors based on histopathology and tumor location would help develop a new therapeutic intervention that may have a clinical impact on prolonging patient survival and improving quality of life. Tumor biology plays a significant role in the prognosis and treatment responses. The tumor biology and biological markers should be understood to develop individualized treatment approaches for the patient’s benefit. For advanced-stage disease or inapproachable tumor location like sphenoid sinus concurrent chemoradiation therapy is favored over surgery with equal results, as complete resection is not possible. Surgery in these cases might increase morbidity with no added benefit results. Single therapy with chemotherapy alone or radiation therapy alone does not have beneficial results as well as compared to combined therapy. Owing to the rarity of SmCC occurrence, it is not easy to conduct a large, randomized control trial to find definite results. Most of the current practice is based on small case series or cases reported that share the treatment approach and outcome in these patients.

## References

[1] Tsoli M, Chatzellis E, Koumarianou A, Kolomodi D, Kaltsas G (2019). Current best practice in the management of neuroendocrine tumors. Ther Adv Endocrinol Metab.

[2] Patel N, Benipal B (2019). Incidence of neuroendocrine tumors in the United States from 2001-2015: a United States cancer statistics analysis of 50 states. Cureus.

[3] Yao JC, Hassan M, Phan A (200820). One hundred years after "carcinoid": epidemiology of and prognostic factors for neuroendocrine tumors in 35,825 cases in the United States. J Clin Oncol.

[4] Choe J, Kim KW, Kim HJ (2019). What is new in the 2017 World Health Organization Classification and 8th American Joint Committee on cancer staging system for pancreatic neuroendocrine neoplasms?. Korean J Radiol.

[5] Wang HY, Zou J, Zhou GY, Yan J-Q, Liu S-X (2014). Primary small cell neuroendocrine carcinoma of the tonsil: a case report and review of the literature. Int J Clin Exp Pathol.

[6] Rekhtman N (2010). Neuroendocrine tumors of the lung: an update. Arch Pathol Lab Med.

[7] Kyriakopoulos G, Mavroeidi V, Chatzellis E, Kaltsas GA, Alexandraki KI (2018). Histopathological, immunohistochemical, genetic and molecular markers of neuroendocrine neoplasms. Ann Transl Med.

[8] Mitchell EH, Diaz A, Yilmaz T (2012). Multimodality treatment for sinonasal neuroendocrine carcinoma. Head Neck.

[9] Walenkamp AM, Sonke GS, Sleijfer DT (2009). Clinical and therapeutic aspects of extrapulmonary small cell carcinoma. Cancer Treat Rev.

[10] Patel TD, Vazquez A, Dubal PM, Baredes S, Liu JK, Eloy JA (2015). Sinonasal neuroendocrine carcinoma: a population-based analysis of incidence and survival. Int Forum Allergy Rhinol.

[11] Pointer KB, Ko HC, Brower JV (2017). Small cell carcinoma of the head and neck: an analysis of the National Cancer Database. Oral Oncol.

[12] Su SY, Bell D, Hanna EY (2014). Esthesioneuroblastoma, neuroendocrine carcinoma, and sinonasal undifferentiated carcinoma: differentiation in diagnosis and treatment. Int Arch Otorhinolaryngol.

[13] Shah K, Perez-Ordóñez B (2016). Neuroendocrine neoplasms of the sinonasal tract: neuroendocrine carcinomas and olfactory neuroblastoma. Head Neck Pathol.

[14] Fitzek MM, Thornton AF, Varvares M (2002). Neuroendocrine tumors of the sinonasal tract. Results of a prospective study incorporating chemotherapy, surgery, and combined proton-photon radiotherapy. Cancer.

[15] van der Laan TP, Iepsma R, Witjes MJ, van der Laan BF, Plaat DE, Halmos GB (2016). Meta-analysis of 701 published cases of sinonasal neuroendocrine carcinoma: the importance of differentiation grade in determining treatment strategy. Oral Oncol.

[16] Faisal M, Haider I, Adeel M (2018). Small cell neuroendocrine carcinoma of nose and paranasal sinuses: the Shaukat Khanum Memorial Cancer Hospital experience and review of literature. J Pak Med Assoc.

[17] Menon S, Pai P, Sengar M (2010). Sinonasal malignancies with neuroendocrine differentiation: case series and review of literature. Indian J Pathol Microbiol.

[18] Bean MB, Liu Y, Jiang R (2019). Small cell and squamous cell carcinomas of the head and neck: comparing incidence and survival trends based on Surveillance, Epidemiology, and End Results (SEER) data. Oncologist.

[19] Hatoum GF, Patton B, Takita C, Abdel-Wahab M, LaFave K, Weed D, Reis IM (2009). Small cell carcinoma of the head and neck: the University of Miami experience. Int J Radiat Oncol Biol Phys.

